# GENERATIVE AI TOWARDS A COMPARATIVE ANALYSIS OF HOSPITALIZATION RATES AMONG PATIENTS WITH ULCERATIVE COLITIS TREATED WITH INFLIXIMAB, VEDOLIZUMAB, AND TOFACITINIB IN THE PUBLIC HEALTHCARE SYSTEM IN BRAZIL: A NATIONWIDE STUDY

**DOI:** 10.1590/S0004-2803.24612025-066

**Published:** 2026-05-22

**Authors:** Douglas Andreas VALVERDE, Abel Botelho QUARESMA, Maria Paz Gimenez VILLAMIL, Thyago Proença de MORAES, Paulo Gustavo KOTZE

**Affiliations:** 1 Techtrials Pesquisa e Tecnologia LTDA, Ciência de Dados, Vinhedo, São Paulo, Brasil.; 2 Universidade do Oeste de Santa Catarina - UNOESC, Cirurgia Colorretal, Joaçaba, SC, Brasil.; 3 Pontifícia Universidade Católica do Paraná, Programa de Pós-Graduação em Ciências da Saúde, Curitiba, Paraná, Brasil.; 4 Pontifícia Universidade Católica do Paraná, Unidade de Cirurgia Colorretal, Ambulatório de DII, Curitiba, Paraná, Brasil.

**Keywords:** Colitis ulcerative, hospitalization, infliximab, vedolizumab, tofacitinib, Colite ulcerativa, hospitalização, infliximabe, vedolizumabe, tofacitinibe

## Abstract

**Objective:**

Ulcerative colitis (UC) is a chronic inflammatory bowel disease that requires advanced therapies, including infliximab, vedolizumab, and tofacitinib. Our aim is to understand the hospitalization rates and outcomes associated with these therapies in real-world scenarios, which is crucial for optimizing patient management and healthcare resource allocation.

**Methods:**

This nationwide retrospective observational study analyzed real-world data (RWD) from Brazil’s public healthcare system (SUS) between January 2021 and December 2023. Data on hospitalizations, length of stay, mortality, costs, and non-IBD-related hospital admissions were extracted and analyzed using the Techtrials TT Disease Explorer and TT RWE Generator, leveraging generative artificial intelligence (GenAI) for data integration and analysis.

**Results:**

A total of 1,313 patients required hospitalization, with 1,434 hospitalizations recorded. The hospitalization rate per 100 patients was highest for tofacitinib (262.5), followed by infliximab (212.88) and vedolizumab (191.67). Patients treated with infliximab had the shortest hospital stay (Median 3.0 days [0-68]) and lowest costs (Median 510.83 BRL [23.45 - 111,594.14]), whilst vedolizumab had the highest hospitalization costs. Surgical intervention rates were highest among tofacitinib users. Non-IBD-related hospitalizations suggested potential adverse drug events, including gastrointestinal disorders, infections, and anemia.

**Conclusion:**

This study represents the first AI-assisted RWE analysis of UC hospitalizations in Brazil. The findings highlight the varying hospitalization burdens across advanced therapies, with tofacitinib associated with higher hospitalization rates and infliximab linked to shorter hospital stays and lower costs. These insights can inform clinical decision-making and healthcare policy. Future studies should further explore AI’s role in optimizing RWE research and UC management.

## HIGHLIGHTS

• Real-world hospitalization rates and outcomes of ulcerative colitis treatments (infliximab, vedolizumab, tofacitinib) were analyzed using Brazilian public health data (2021-2023).

• Tofacitinib showed the highest hospitalization rate, longer stays, and higher colectomy rates, indicating more severe cases.

• Infliximab was linked to shorter hospital stays and lower costs.

• Vedolizumab had the highest costs due to multiple admissions.

• Adverse events, such as gastrointestinal disorders and infections, were observed, emphasizing the need for careful monitoring.

• Artificial intelligence powered the analysis, showcasing its role in improving medical research, evidence-based medicine, and resource planning.

## Resumo

**Objetivo:**

A retocolite ulcerativa (RCU) é uma doença inflamatória intestinal crônica que requer terapias avançadas, incluindo infliximabe, vedolizumabe e tofacitinibe. Nosso objetivo é compreender as taxas de hospitalização e os desfechos associados a essas terapias em cenários de mundo real, o que é fundamental para otimizar o manejo dos pacientes e a alocação de recursos em saúde.

**Métodos:**

Este estudo observacional retrospectivo de âmbito nacional analisou dados de mundo real (RWD) do sistema único de saúde (SUS) do Brasil entre janeiro de 2021 e dezembro de 2023. Dados sobre hospitalizações, tempo de internação, mortalidade, custos e admissões hospitalares não relacionadas à DII foram extraídos e analisados utilizando o Techtrials TT Disease Explorer e o TT RWE Generator, com o uso de inteligência artificial generativa (GenAI) para integração e análise dos dados.

**Resultados:**

Um total de 1.313 pacientes necessitou de hospitalização, com 1.434 internações registradas. A taxa de hospitalização por 100 pacientes foi mais alta para tofacitinibe (262,5), seguida por infliximabe (212,88) e vedolizumabe (191,67). Pacientes tratados com infliximabe apresentaram o menor tempo de internação (mediana de 3,0 dias [0-68]) e os menores custos (mediana de R$ 510,83 [23,45 - 111.594,14]), enquanto o vedolizumabe apresentou os maiores custos de hospitalização. As taxas de intervenção cirúrgica foram mais elevadas entre os usuários de tofacitinibe. As hospitalizações não relacionadas à DII sugeriram potenciais eventos adversos aos medicamentos, incluindo distúrbios gastrointestinais, infecções e anemia.

**Conclusão:**

Este estudo representa a primeira análise de RWD sobre hospitalizações por RCU no Brasil com o auxílio de IA. Os resultados destacam diferentes impactos das terapias avançadas nas hospitalizações, com tofacitinibe associado a maiores taxas de hospitalização e infliximabe ligado a menores tempos de internação e custos. Essas informações podem subsidiar tomadas de decisão clínica e políticas de saúde. Estudos futuros devem explorar mais profundamente o papel da IA na otimização da pesquisa em RWD e no manejo da RCU.

## INTRODUCTION

Inflammatory bowel diseases (IBD) encompass a group of chronic inflammatory conditions of the gastrointestinal tract, primarily including Crohn’s disease (CD) and ulcerative colitis (UC). UC is characterized by continuous mucosal inflammation starting in the rectum and extending proximally through parts of the colon. The symptoms of UC can significantly impact the quality of life, presenting with persistent diarrhea, abdominal pain, rectal bleeding, and weight loss. The exact etiology of UC remains unclear, but it is believed to result from an abnormal immune response in genetically predisposed individuals, possibly triggered by environmental factors^1^.

The management of UC involves a range of therapeutic strategies aimed at inducing and maintaining remission, reducing inflammation, and preventing complications^1^. Traditional treatments include aminosalicylates, corticosteroids, and immunomodulators. However, the introduction of biological agents and small molecules has revolutionized the treatment landscape for moderate to severe UC^2^ ^,^ ^3^. Advanced therapies such as infliximab, vedolizumab, and tofacitinib block specific components of the immune system, offering more targeted and effective therapy options^4^. All these three therapies are available in the public healthcare system in Brazil as options to patients with UC^5^.

Approximately one-fifth of patients with IBD will experience hospitalization at some point during the course of the disease^6^. Studies analysing hospitalization rates report distinct trends: some indicate a reduction, others show stabilization, while some point to an increase across various regions worldwide^6^ ^-^ ^8^. Generally, these hospitalizations involve patients with more severe manifestations of UC, who do not respond adequately to proposed treatments. This scenario not only intensifies the complexity of clinical management but also significantly increases the total costs associated with care, impacting both public and private health services^9^ ^,^ ^10^.

The increasing availability of real-world data (RWD) from electronic health records, administrative databases, and patient registries offers valuable insights into the effectiveness and safety of treatments in routine clinical practice. In Brazil, the unified health system (sistema único de saúde, SUS) provides comprehensive healthcare to the population. DATASUS, the department of informatics of SUS, manages health information and data, including hospitalizations, treatments, and outcomes^11^. Leveraging data from SUS and DATASUS, Techtrials’ platform integrates and analyzes health data to support healthcare research and decision-making. In the IBD field, this automated data extraction platform was described in a recent large epidemiology study^12^. Additionally, it was featured in a more recent study that analysed temporal trends in surgeries for CD in Brazil^13^.

The application of artificial intelligence is becoming more common in data analysis in medical sciences. The use of technology to capture and analyze data saves time from researchers and can optimize resources as a consequence to fast generating results^14^ ^-^ ^17^. Techtrials is a leading health data analytics company that provides a comprehensive platform for accessing and analyzing real-world healthcare data. The Techtrials platform stands out due to its advanced algorithms that clean and standardize data, ensuring high consistency and accuracy. By integrating vast datasets from multiple sources, including hospitalizations, outpatient procedures, drug utilization, and geographic data, the platform provides a comprehensive overview of a current disease. Additionally, it supports advanced statistical analysis such as regression models, survival analysis, and comparative effectiveness research, which enhances the depth and reliability of the analysis. Key features of the Techtrials platform include data integration, cleaning and standardization, advanced analytics, with a user-friendly interface generating customizable reports with compliance and security. After automated data capturing, the platform is linked to a specific RWE generator, which leverages GenAI to facilitate complex study designs. This generator enhances data with complete analyses, generating real world evidence (RWE) data from specific databases^12^ ^,^ ^13^.

This study aimed to demonstrate the first real-world evidence (RWE) GenAI data in the management of UC in Brazil. We specifically aimed to compare hospitalization rates between patients treated with infliximab, vedolizumab, and tofacitinib. Additionally, secondary outcomes such as length of hospital stay, mortality rates and costs were also analyzed and compared between the three different types of advanced therapies. Lastly, surgery rates and non-IBD related hospitalizations were identified.

## METHODS

### Type of study and population

The study was a retrospective, RWE, observational analysis that included all patients diagnosed with ulcerative colitis (ICD code K51 and variants) treated with advanced therapies reimbursed by the Brazilian unique national public helthcare (sistema único de saúde - SUS). Specifically, it focused on infliximab (IFX), vedolizumab (VEDO), and tofacitinib (TOFA). It considered those hospitalized in the public healthcare system between January 2021 and December 2023.

### Database and outcomes

Data was collected automatically from the DATASUS database by the platform TT Disease Explorer, from Techtrials Pesquisa e Tecnologia Ltda. (São Paulo, Brazil). After data collection, patients were classified in three groups according to the type of advanced therapies used. Hospitalizations were captured and rates were compared between the three agents. Secondary information as length of hospital stay, costs and mortality were additionally analyzed. Non-IBD related hospitalizations in these patients were also described. After data was extracted from TT Disease Explorer it was submitted to TT RWE Generator and the authors used this GenAI agent to discuss possible study designs, statistical analysis planning, followed by initial manuscript writing. After continuing iteration with TT RWE Generator the authors collected all the material generated by the GenAi-Author discussion. This initial document with text, tables, calculations and charts was used as the main base for the current study. All analyses generated by the GenAI methodology were checked by one of the authors (TPM), with STATA software v. 18 (College Station, Texas, United States) to avoid bias.

### Original datasets

The data used in this study were obtained from two publicly available datasets: drug utilization from SIASUS (contains records of drug utilization by patients over time) and hospitalizations from SIHSUS (contains hospitalization records, including patient ID, ICD description, procedures, length of stay, costs, and outcomes)**.**

### TT RWE Generator

This study was performed by using TT RWE Generator, an advanced GenAi agent created to receive data from the Techtrials platform. The TT RWE Generator contains specific core instructions that leverages the power of GenAI to facilitate complex and reliable study designs, ensuring robust support for healthcare research and decision-making. Key benefits of the TT RWE Generator include automated study design (PICOT methodology), verification of data consistency across sources, alignment of geographic identifiers like cities and municipalities, and checking of data formatting to support accurate comparisons and matches across datasets. It provides expert support, perform data analysis, complex statistical calculations and maintains academic rigor, aligning with NICE^18^ and EMA^19^ frameworks and offering practical, professional insights on using RWD in healthcare research^20^.

### Data cleaning and filtering

The datasets were merged using the patients’ anonymous IDs. This allowed for a comprehensive dataset where hospitalization records were linked with drug usage history for each patient. To ensure the analysis only included relevant data, we filtered the records to include only hospitalizations that occurred after the start of drug usage. This filtering step was crucial to accurately assess the impact of each drug on hospitalization rates and outcomes.

### Statistical plan and analysis

### Descriptive analysis

We began with a descriptive analysis to summarize the key characteristics of the dataset. This included calculating the mean, median, standard deviation, and interquartile range for continuous variables such as age, length of stay, and hospitalization costs. Categorical variables such as drug type and ICD descriptions were summarized using frequency and percentage distributions.

Assumptions made during the statistical analysis include the normality of continuous variables and homogeneity of variances across groups. These assumptions were verified using statistical tests such as the Shapiro-Wilk test for normality and Levene’s test for equality of variances. Any deviations from these assumptions were addressed using appropriate non-parametric tests or transformations.

### Comparison of hospitalization rates

To compare the hospitalization rates among the three drugs (infliximab, vedolizumab, and tofacitinib), we calculated the total number of hospitalizations, and the number of unique patients hospitalized for each drug. The hospitalization rate per 100 patients was then derived by normalizing the total hospitalizations against the number of patients.

### Analysis of secondary outcomes

Secondary outcomes, including the length of hospital stay, mortality rates during admission, and hospitalization costs, were analyzed using the following methods:

**Length of hospital stay**: We calculated the average length of stay for each drug and performed a Kruskal-Wallis test to determine if there were significant differences between the groups.

**Mortality rates**: Death rates were compared using chi-square tests to assess if there were significant differences in mortality between the drug groups. The study focused on mortality rates only during hospitalization, ensuring that the data directly reflected the immediate impact of the treatments, free from external influences post-discharge. This approach ensured a precise and relevant analysis of therapeutic efficacy under controlled conditions.

**Hospitalization costs**: We analyzed the average costs of hospitalization for each drug using a Kruskal-Wallis test, given the non-normal distribution of cost data.

### Analysis of non-IBD related hospitalizations

To identify non-IBD related hospitalizations, we excluded hospitalizations with ICD codes related to IBD (K50 and K51). The remaining hospitalizations were then categorized and analyzed to determine the most common causes and their associated outcomes. We compared the frequency, length of stay, and costs of these hospitalizations between the three different study groups (infliximab, vedolizumab and tofacitinib).

### Ethical considerations

This study was approved by the Research Ethics Committee of the Pontifical Catholic University of Paraná (PUCPR), according to the Certificate of Presentation for Ethical Assessment (CAAE) 59111522.8.0000.0020. During all stages of this research, all guidelines of Resolution 466/2012 of the National Health Council were strictly followed and complied with.

## RESULTS

During the study period, a total of 1,313 patients required hospital admissions, with a total of 1,434 hospitalizations being recorded in the national public healthcare system among patients with UC who used any of the three types of advanced therapies. The length of stay in hospital varied from 0 to 68 days, with costs ranging from BRL 21.98 to 111,594.14. 

### Hospitalization rates per agent

The total number of hospitalizations and the number of unique patients using each drug are summarized in Table 1. As seen, patients using tofacitinib had proportionally higher rates of hospitalizations. These calculations only include hospitalizations occurring after the start of each respective drug. 

**TABLE 1:** Total of hospitalizations per advanced therapy with hospitalization rates.

Drug	Total hospitalizations	Unique patients using therapy	Hospitalization rate per 100 patients
Infliximab	909	427	212.88
Vedolizumab	483	252	191.67
Tofacitinib	42	16	262.50

### Secondary outcomes

**FIGURE 1:**
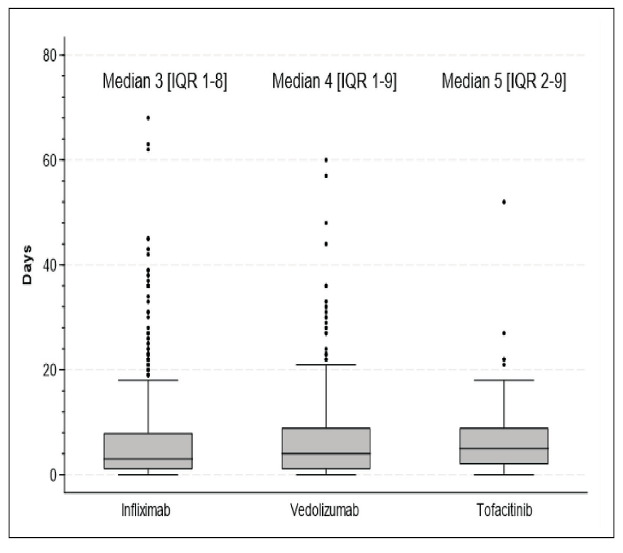
Median length of stay per agent. Infliximab vs tofacitinib (*P*=0.01), Infliximab vs Vedolizumab (*P*=0.03), tofacitinib vs vedolizumab (*P*=0.11). Kruskal Wallis test.

**FIGURE 2:**
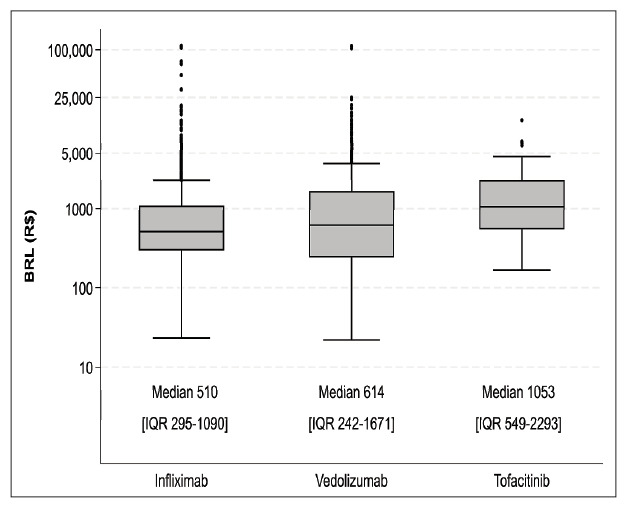
Median costs per agent. Infliximab vs tofacitinib(*P* <0.001), infliximab vs vedolizumab (*P*<0.01), tofacitinib vs vedolizumab (*P* =0.05) - Kruskal Wallis

Table 2 provides a detailed breakdown of length of hospital stay, mortality rates during hospitalization, and hospitalization costs for each type of advanced therapy. Hospitalizations associated with infliximab had shorter average stays, while no mortality was recorded in the tofacitinib group (Figure 1) Costs tended to be higher in the vedolizumab group (Figure 2).

**TABLE 2:** Lenght of stay mortality and costs os hospitalizations per advanced therapy.

	Length of hospital stay	Mortality rates during hospitalization	Hospitalization costs
Infliximab (N=909)	Mean 5.98±8.46, Median 3.0 (0-68)	16 (1.76%)	Mean 1,839.89±7,573.77, Median 510.83 (23.45-111,594.14)
Vedolizumab (N=483)	Mean 6.71±8.46, Median 4.0 (0-60)	14 (2.90%)	Mean 2,229.02±7,480.41, Median 614.86 (21.98-110,692.21)
Tofacitinib (N=42)	Mean 8.24±9.67, Median 5.0 (0-52)	0 (0%)	Mean 1,933.91±2,465.11, Median 1,052.81 (167.15-12,972.25)

### Analysis of patients using multiple drugs (polytherapy)

During the study period, some patients used more than one advanced therapy, either sequentially or overlapping, and were hospitalized after starting these treatments. Overall, 59 patients used more than one drug during the study period and 185 unique hospitalizations were recorded among these patients. Infliximab was associated with the highest number of hospitalizations (160) among polytherapy patients, with 53 unique patients. From the remaining cases, 112 included vedolizumab in 55 patients, and 34 included tofacitinib in only 11 patients.

### Detailed analysis of causes of non-ibd hospitalizations and possibility of adverse drug events

To assess non-IBD hospitalizations and identify potential adverse drug events (ADEs), we examined hospitalizations with ICD codes unrelated to IBD. This analysis focused on the top 20 most frequent non-IBD ICD codes associated with each drug. Each ICD code represents hospitalizations possibly triggered by conditions that may relate to ADEs from advanced therapies used in UC treatment. Table 3 lists the top 20 non-IBD ICD codes, specifying the number of unique hospitalizations and patients per ICD code and drug:

**TABLE 3:** Most common ICD codes for non-IBD related admissions.

ICD Code description	Drug	Occurrences	Unique patients
K92.9 Disease of digestive system, unspecified	Infliximab	98	19
K92.8 Other specified diseases of digestive system	Infliximab	26	21
K63.8 Other specified diseases of intestine	Infliximab	18	15
C83.5 Lymphoblastic (diffuse) non-Hodgkin’s lymphoma	Vedolizumab	15	1
B34.2 Coronavirus infection, unspecified	Infliximab	10	9
K92.8 Other specified diseases of digestive system	Vedolizumab	10	9
D64.9 Anemia, unspecified	Infliximab	9	9
K63.9 Disease of intestine, unspecified	Infliximab	9	8
O80.0 Spontaneous vertex delivery	Infliximab	9	7
A09 Infectious gastroenteritis and colitis, unspecified	Infliximab	8	8
A49.9 Bacterial infection, unspecified	Infliximab	8	7
K93.8 Disorders of other specified digestive organs	Infliximab	7	7
Z94.4 Liver transplant status	Infliximab	7	2
A49.8 Other bacterial infections of unspecified site	Vedolizumab	6	6
K63.8 Other specified diseases of intestine	Vedolizumab	6	6
K83.0 Cholangitis	Vedolizumab	6	4
A41.9 Sepsis, unspecified organismo	Infliximab	5	5
D50.9 Iron deficiency anemia, unspecified	Infliximab	5	4
J18.9 Pneumonia, unspecified organismo	Infliximab	5	5
J18.9 Pneumonia, unspecified organismo	Vedolizumab	5	5

### Analysis and potential adverse drug events (ADEs)

Each ICD code was examined by the TT REW Generator to identify possible ADEs, and below are the AI considerations made:

Digestive and gastrointestinal issues (e.g., K92.9, K92.8, K63.8) were frequently reported for infliximab and vedolizumab users, suggesting potential gastrointestinal ADEs.

Anemia (D64.9 and D50.9) and bacterial infections (A49.9, A49.8) may be related to immunosuppression, which is a known effect of immunomodulatory therapies.

Non-Hodgkin’s lymphoma (C83.5), though rare, appeared in a patient with vedolizumab therapy, which could warrant additional monitoring.

### Intestinal surgery rates analysis

Table 4 describes the number of patients submitted to surgical procedures in each group. As seen, 52 patients under infliximab therapy underwent 64 procedures, 42 patients with vedolizumab underwent 45 procedures and 6 patients using tofacitinib were submitted to 9 operations. Surgical rates were higher in tofacitinib patients, followed by infliximab and vedolizumab.

**TABLE 4:** Surgery rates per agent.

Drug	Total surgeries	Unique patients	Surgery rate per 100 patients
Infliximab	64	52	123.08
Vedolizumab	45	42	107.14
Tofacitinib	9	6	150.00

## DISCUSSION

In this generative artificial intelligence derived retrospective observational nationwide study, proportional hospitalization rates with publicly reimbursed advanced therapies infliximab, vedolizumab and tofacitinib were 212.88, 191.67 and 262.50 per 100 patients, respectively. Mean length of hospital stay (in days) was longer with tofacitinib, followed by vedolizumab and infliximab. Hospitalization costs were higher with vedolizumab, followed by tofacitinib and infliximab. Several non-IBD related causes of hospitalizations suggested potential adverse events related to the drugs.

This is the first study in the IBD field which used generative artificial intelligence (GenAI) for both data preparation, and analysis. Moreover, the platform also supported manuscript writing. By utilizing the TT RWE Generator, we were able to efficiently integrate diverse datasets, apply statistical planning, and generate comprehensive reports. This integration significantly enhanced the accuracy and reliability of our real-world evidence study on hospitalization rates among different treatments for UC in Brazil. Data extraction from the publicly available database^11^ was previously used in the largest epidemiology IBD Brazilian study to date^12^, among other studies in the oncology field^21^. In the present study, we integrated data extraction with data analysis and manuscript ordination and writing, what can help researchers by reducing time of drafting and enhancing AI-based quality on analyses.

Our results demonstrated that between January 2021 to December 2023, hospitalizations with infliximab were more commonly observed in the public setting in Brazil, followed by vedolizumab and tofacitinib. This possibly occurs as infliximab is the most prescribed agent in the management of moderate to severe UC. Numbers with tofacitinib were lower, as this oral agent was approved more recently in the treatment of UC in our country. With all agents, the number of hospitalizations was higher than the number of unique patients using the drug. This demonstrates that some patients had more than one hospitalization during the study period. Proportionally, hospitalization rates were higher with tofacitinib (262.5/100 patients) in comparison with infliximab (212.88/100 patients) and vedolizumab (191.67/100 patients). This can possibly be explained by the fact that some patients may have failed previously infliximab or vedolizumab, using tofacitinib as a last therapeutic option, with more severe cases leading to more hospital admissions. Length of stay in hospital was also longer with tofacitinib, what can be explained by this possible multi-failed population, as previously discussed. Colectomy rates also tended to be higher in the tofacitinib group, what emphasizes this explanation.

Mean length of stay ranging from 3-5 days is probably related to potential adverse events, and surgery may extend duration of hospitalization due to possible complications. Colectomy rates per 100 patients were 123.08, 107.14 and 150 with infliximab, vedolizumab and tofacitinib, respectively. As tofacitinib^22^ ^-^ ^24^ and infliximab^25^ ^-^ ^26^, can be used as rescue therapies in cases of acute severe it was expected that surgery would be more common in these groups in comparison with vedolizumab, which is not used in the acute scenario. If these surgical procedures were performed in emergency or elective setting, this can only be speculated, as the platform cannot capture the regimen of surgery.

Mean hospitalization-related costs were higher with vedolizumab in comparison with the two other agents. We believe this finding can be explained by 15 hospital admissions observed on one unique patient using vedolizumab with a lymphoblastic non-Hodgkin’s lymphoma, possibly related to chemotherapy or complications of the therapy. Despite that unique situation, costs were not so different between the therapies, emphasizing the complexity of patients with moderate to severe UC when admitted to the hospital. 

Mortality during hospitalizations was observed in 16 patients using infliximab (1.76%) and 14 with vedolizumab (2.9%). No deaths were observed in the tofacitinib group, possibly due to the reduced number of patients in this group. Mortality can be related to several possible causes, including colectomy or infections due to systemic immunosuppression. Precise causes of death in these patients could not be retrieved by the platform used in data extraction and comprise one of the inherent limitations of this methodology.

Some of the included patients used polytherapy, with more than one of the agents, during the study period. This polytherapy group may provide valuable insights into complex cases where patients possibly do not respond adequately to a single therapy. Details of sequencing of agents during the study period could also not be retrieved by the platform in DATASUS^11^. As infliximab is probably used as first line throughout the country, due to the long experience with this agent from physicians, most patients in polytherapy were observed in the infliximab group (160 of the 185 unique hospitalizations captured).

One of the most notable features of the platform was the data extraction and analysis on causes of hospitalizations, and their possible correlation to potential adverse events, performed by GenAI. It is noteworthy that despite the non-ICD codes fulfilled in admissions, some generic codes could be related to digestive symptoms, caused by the UC itself or a flare (e.g. K92.9 Disease of digestive system, unspecified; K92.8 Other specified diseases of digestive system; K63.8 Other specified diseases of intestine). These unspecific ICD codes were responsible for the majority of admissions. Infections, such as COVID-19, sepsis, cholangitis and pneumonia, were also observed in a significant proportion of patients, mostly using infliximab (maybe also related to the fact that this agent is mostly used in combination with immunomodulators). Other possible potential adverse events such as anemia, could also be observed. 

The present study is associated to some limitations. First, the variables captured by the platform did not include some trivial information, such as age at diagnosis, extension of the disease among others. If surgical rates could be related to urgent or elective procedures, this could also not be captured. In cases of polytherapy, sequencing of the agents could not also be retrieved, limiting information in these patients using more than one agent during the study period. Hospitalizations were only retrieved in the public setting, not including patients with health insurance from the private healthcare system. Despite these limitations, our study has some strengths. First, as far as to our knowledge, this is the first automated GenAI extracted and generated dataset in the field of IBD. Despite the platform for data extraction was used previously in our national database, the RWE generator platform used to generate analysis and manuscript drafting is innovative in our field. All analyses were checked manually by a statistician, what confers reproducibility. To use GenAI tools as a guidance to facilitate writing and reduce time for drafting documents will probably become a trend in the future. We believe this study represents a step forward the use of GenAI in IBD datasets throughout the globe and adds value to the literature. More importantly, this is the most robust data on hospitalizations in UC in Brazil in the biologic era in the public setting and deserves attention from public authorities when planning health resources for the future.

This study not only contributes to the growing body of evidence on treatment outcomes in IBD but also showcases the transformative potential of AI in healthcare research. As AI models such as OpenAi Deep Research continue to evolve, we are excited about their potential to refine study methodologies, optimize treatment strategies, and generate even more precise and impactful medical insights. The future of AI-driven healthcare research is promising, and we look forward to further advancements that will improve patient outcomes and shape the next era of evidence-based medicine.

## CONCLUSION

This pioneering study used generative artificial intelligence to analyze data from hospitalized patients with ulcerative colitis in the Brazilian public healthcare system, showing that hospitalization rates with infliximab, vedolizumab, and tofacitinib were 212.88, 191.67, and 262.50 per 100 patients, respectively. Tofacitinib showed the highest rates and was associated with longer hospital stays and higher colectomy rates, reflecting more severe cases. Patients who used infliximab had shorter hospital stays and lower costs, while vedolizumab registered the highest hospitalization costs due to an exceptional case of multiple admissions. Hospitalizations for non-IBD-related reasons suggested possible adverse events related to the medications. This innovative AI-driven approach demonstrates the potential to transform and optimize medical research, bringing new perspectives for improving patient outcomes in evidence-based medicine and highlighting the expectation of even more detailed data from generative artificial intelligence tools in the IBD field.

## Data Availability

Data-available-upon-request
